# CYP2C19 Loss‐of‐Function Is an Independent Risk Factor of Coronary Artery Disease in Patients With Hypertension

**DOI:** 10.1002/jcla.70088

**Published:** 2025-07-25

**Authors:** Guoliang Wei, Bin Li, Hao Wang, Wenhao Chen, Kehui Chen, Weihong Wang, Shen Wang, Hui Zeng, Yuanliang Liu, Yue Zeng, Hui Rao

**Affiliations:** ^1^ Center for Cardiovascular Diseases Meizhou People's Hospital, Meizhou Academy of Medical Sciences Meizhou China; ^2^ Department of Laboratory Medicine Meizhou People's Hospital, Meizhou Academy of Medical Sciences Meizhou China

**Keywords:** coronary artery disease, *CYP2C19*, hypertension population, polymorphism

## Abstract

**Objective:**

Cytochrome P450 2C19 (CYP2C19) is affected by its gene polymorphisms and is involved in the occurrence and development of diseases. To assess the relationship between *CYP2C19* polymorphisms and coronary artery disease (CAD) susceptibility in hypertensive patients.

**Methods:**

This study retrospectively analyzed 3404 hypertensive patients who were admitted to Meizhou People's Hospital from November 2019 to August 2023, including 1438 CAD patients and 1966 nonCAD individuals. The *CYP2C19* rs4244285 (681G>A, *2) and rs4986893 (636G>A, *3) polymorphisms were genotyped by polymerase chain reaction (PCR)‐chip technique. The relationship between *CYP2C19* polymorphisms and CAD was analyzed.

**Results:**

There were 1567 (46.0%), 1491 (43.8%), and 346 (10.2%) individuals with *CYP2C19* extensive metabolizer (EM) (CYP2C19*1/*1), intermediate metabolizer (IM) (CYP2C19*1/*2 and *1/*3), and poor metabolizer (PM) (CYP2C19*2/*2, *2/*3, and *3/*3) phenotype. The CAD patients had higher frequencies of the *2 allele (30.2% vs. 26.0%, *p* < 0.001), *3 allele (4.9% vs. 3.8%, *p* = 0.021) and lower frequency of *1 allele (64.8% vs. 70.2%, *p* < 0.001) than controls. Logistic regression analysis showed that body mass index (BMI) ≥ 24.0 kg/m^2^ (odds ratio (OR): 1.364, 95% confidence interval (CI): 1.184–1.571, *p* < 0.001), history of alcoholism (OR: 1.761 95% CI: 1.211–2.559, *p* = 0.003), and *CYP2C19* IM + PM phenotypes (IM + PM vs. EM, OR: 1.314, 95% CI: 1.145–1.508, *p* < 0.001) were independent risk factors for CAD in hypertensive patients.

**Conclusions:**

*CYP2C19* loss‐of‐function, BMI ≥ 24.0 kg/m^2^, and history of alcoholism were independent risk factors for CAD in hypertensive patients. Hypertensive patients who carried *CYP2C19* loss‐of‐function need to be aware of the risk of developing CAD, and it provides further evidence for the relationship between CYP2C19 and CAD.

## Graphical abstract

The relationship between Cytochrome P450 2C19 (CYP2C19) polymorphisms and coronary artery disease (CAD) in hypertensive patients was analyzed. *CYP2C19* loss‐of‐function, BMI ≥ 24.0 kg/m^2^, and history of alcoholism were independent risk factors for CAD in hypertensive patients. Hypertensive patients who carried *CYP2C19* loss‐of‐function need to be aware of the risk of developing CAD, and it provides further evidence for the relationship between CYP2C19 and CAD.

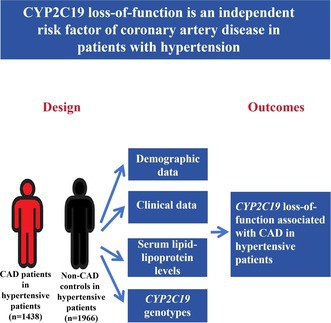

## 1 Introduction

Hypertension is a major disease burden, characterized by a sustained increase in systolic blood pressure (SBP) and/or diastolic blood pressure (DBP) of the systemic arteries [1]. Hypertension has become one of the main public health problems in China [2]. The standardized prevalence of hypertension in adults aged 18–69 in China is about 24.7% [3]. More than 70% of cardiovascular diseases (CVDs) cases and deaths are attributable to modifiable risk factors, with hypertension being the most important risk factor, with a population attributable fraction (PAFs) of 22.3% [4].

Hypertension is strongly associated with an increased risk of coronary artery disease (CAD). CAD is a heart disease caused by myocardial ischemia or necrosis due to stenosis of coronary artery atherosclerosis [5], it is one of the major public health problems. A cohort study showed that the risk of death from CAD increased by 26% for every 20 mmHg increase in unadjusted systolic blood pressure (SBP) [6]. The prevalence rate of hypertension combined with CAD is increasing year by year [7]. Hypertension and CAD can coexist in the same person, and individuals with hypertension are more likely to develop CAD than those with normal blood pressure [8]. When hypertension is combined with CAD, the treatment of both diseases presents additional challenges. Therefore, predicting the risk of CAD in hypertensive patients may be more beneficial to the treatment and control of these diseases [9].

Cytochrome P450 (CYP450) enzymes are involved in the biotransformation and metabolism of substances [10], and the pathophysiological processes of various diseases [11]. CYP450 2C19 (CYP2C19) is an important member of the CYP450 family; it not only participates in the metabolism of various drugs [12, 13], but also plays a key role in the metabolism of endogenous substances such as arachidonic acid and fatty acids [14]. Abnormal metabolism of these substances is closely related to pathological processes such as vascular endothelial function impairment and the formation of atherosclerotic plaques [15, 16]. In the pathogenesis of CVDs, the interaction between genetic factors and environmental factors is one of the important research topics [17, 18]. Genetic polymorphisms, as an important marker of genetic susceptibility, have been confirmed to be closely related to the risk of various CVDs [19, 20]. However, for a specific group of people with hypertension, there is currently no systematic conclusion regarding how the polymorphisms of the *CYP2C19* gene increase the risk of CAD by influencing metabolic pathways, inflammatory responses, or vascular remodeling [21, 22]. Moreover, hypertension itself promotes the occurrence of CAD by increasing blood pressure and damaging the vascular endothelium, while whether *CYP2C19* gene polymorphisms interact with the pathological and physiological processes of hypertension to further amplify or weaken the risk of CAD remains a scientific issue that needs to be clarified in current research.

CYP2C19 there are two major single‐nucleotide polymorphisms (SNPs) rs4244285 (681G>A, *2) and rs4986893 (636G>A, *3) in *CYP2C19*. Based on the SNPs, CYP2C19 can be divided into six genotypes (*1/*1, *1/*2, *1/*3, *2/*2, *2/*3, and *3/*3) and three phenotypes (extensive metabolizer (EM) (*1/*1), intermediate metabolizer (IM) (*1/*2 and *1/*3), and poor metabolizer (PM) (*2/*2, *2/*3, and *3/*3)) [23]. CYP2C19*2 and *3 are known as CYP2C19 loss‐of‐function alleles [24]. Hokimoto et al. found that CYP2C19 PM was a risk factor for CAD in Japanese women [25]. Shi et al. suggested that the CYP2C19 EM phenotype was dominant in CAD patients among a Chinese population [21]. However, in the specific population with hypertension, is there still an association between CYP2C19 gene polymorphism and susceptibility to CAD? This research aims to explore this scientific issue.

## 2 Materials and Methods

### 2.1 Study Participants and Data Collection

This study retrospectively analyzed 3404 hypertensive patients who were admitted to Meizhou People's Hospital from November 2019 to August 2023. Among the 3404 hypertensive patients, 1438 CAD patients served as the study group and 1966 nonCAD patients with hypertension served as the control group. This study was performed under the guidance of the Declaration of Helsinki and approved by the Ethics Committee of Medicine, Meizhou People's Hospital (Clearance No.: 2024‐C‐17).

The diagnostic criteria for hypertension: without the use of antihypertensive drugs, three measurements of blood pressure on different days show that a mean SBP > 140 mmHg and/or a mean DBP > 90 mmHg [26]. If the patient has a history of hypertension and is currently taking antihypertensive medications, even if the blood pressure measurement is normal, they should still be regarded as hypertensive patients. The diagnostic criteria for CAD: coronary angiography (CAG) showed that at least one of the main epicardial vessels (including left main branch, anterior descending branch, circumflex branch, and right coronary artery) had a diameter stenosis > 50%, and clinically diagnosed myocardial infarction [27]. Information such as age, sex, body mass index (BMI), history of smoking, history of alcohol consumption, history of diabetes mellitus, and history of hypertension was collected from the patient's medical record information system. BMI was divided into three grades: < 18.5, 18.5–23.9, and ≥ 24.0 kg/m^2^.

### 2.2 Determination of Serum Lipids and *CYP2C19* Genotyping

Fasting blood was collected and serum was isolated. Total cholesterol (TC), triglyceride (TG), high‐density lipoprotein cholesterol (HDL‐C), low‐density lipoprotein cholesterol (LDL‐C), apolipoprotein A1 (Apo‐A1), and apolipoprotein B (ApoB) levels in serum samples were assessed using an automatic biochemical analysis system (Olympus AU5400 system, Tokyo, Japan) and corresponding kits.

Genomic DNA was extracted from venous blood collected from EDTA anticoagulant collection vessels using a blood DNA isolation kit (Qiagen GmbH, Germany). The quality and concentration of the DNA were assessed using a Nano‐Drop 2000 spectrophotometer (ThermoFisher Scientific, USA). CYP2C19*2 and CYP2C19*3 variant alleles were genotyped by a *CYP2C19* genotyping kit (BaiO Technology Co Ltd., Shanghai, China).

### 2.3 Statistical Analysis

All statistical analyses were performed using SPSS statistical software version 26.0 (IBM Inc., USA). Continuous variables were expressed as means ± standard deviations and were compared using either Student's *t*‐test or the Mann–Whitney *U* test. The Hardy–Weinberg equilibrium analysis of the subjects, and the comparison of genotype composition ratio and allele frequency between the two groups were analyzed using *χ* ^2^ test. Univariate analysis and multivariate logistic regression analysis were applied to examine the relationship between *CYP2C19* metabolic phenotypes and CAD in patients with hypertension. *p* < 0.05 was considered to represent statistical significance.

## 3 Results

### 3.1 Characteristics of Subjects

Among the hypertensive patients in this study, 2115 (62.1%) were men and 1289 (37.9%) were women. There were 1176 (34.5%) patients who were < 65 years old and 2228 (65.5%) patients who were ≥ 65 years old. There were no significant differences in gender distribution and age distribution between CAD patients and controls (all *p* > 0.05). In the controls, there were 67 (3.4%), 984 (50.1%), and 915 (46.5%) patients with BMI < 18.5 kg/m^2^, 18.5–23.9 kg/m^2^, and ≥ 24.0 kg/m^2^, respectively. There were 42 (2.9%) CAD patients with BMI < 18.5 kg/m^2^ and 769 (53.5%) CAD patients with BMI ≥ 24.0 kg/m^2^. The proportion of BMI ≥ 24.0 kg/m^2^ in CAD patients was higher than that in controls (53.5% vs. 46.5%, *p* < 0.001). The proportion of history of alcohol consumption in CAD patients was higher than that in controls (5.1% vs. 2.9%, *p* = 0.001). There was no significant difference in proportions of history of smoking and diabetes mellitus between CAD patients and controls (all *p* > 0.05). The CAD patients had higher TC (4.59 ± 1.27 vs. 4.38 ± 1.04 mmol/L, *p* < 0.001), TG (1.86 ± 1.45 mmol/L vs. 1.63 ± 1.02 mmol/L, *p* < 0.001), and lower HDL‐C (1.23 ± 0.33 mmol/L vs. 1.29 ± 0.34 mmol/L, *p* < 0.001) and Apo‐A1 (1.13 ± 0.26 g/L vs. 1.16 ± 0.28 g/L, *p* = 0.003) levels than controls (Table 1).

**TABLE 1:** Clinical characteristics of the subjects of this study.

Variables	Total (*n* = 3404)	Controls (*n* = 1966)	CAD patients (*n* = 1438)	*p*
Gender				
Male, *n* (%)	2115 (62.1%)	1232 (62.7%)	883 (61.4%)	0.474
Female, *n* (%)	1289 (37.9%)	734 (37.3%)	555 (38.6%)	
Age, years				
< 65, *n* (%)	1176 (34.5%)	668 (34.0%)	508 (35.3%)	0.422
≥ 65, *n* (%)	2228 (65.5%)	1298 (66.0%)	930 (64.7%)	
BMI (kg/m^2^)				
< 18.5	109 (3.2%)	67 (3.4%)	42 (2.9%)	< 0.001
18.5–23.9	1611 (47.3%)	984 (50.1%)	627 (43.6%)	
≥ 24.0	1684 (49.5%)	915 (46.5%)	769 (53.5%)	
History of smoking				
No	2694 (79.1%)	1579 (80.3%)	1115 (77.5%)	0.050
Yes	710 (20.9%)	387 (19.7%)	323 (22.5%)	
History of alcohol consumption				
No	3273 (96.2%)	1909 (97.1%)	1364 (94.9%)	0.001
Yes	131 (3.8%)	57 (2.9%)	74 (5.1%)	
Diabetes mellitus				
No	2403 (70.6%)	1387 (70.5%)	1016 (70.7%)	0.970
Yes	1001 (29.4%)	579 (29.5%)	422 (29.3%)	
Serum lipid‐lipoprotein levels				
TC, mmol/L	4.47 ± 1.15	4.38 ± 1.04	4.59 ± 1.27	< 0.001
TG, mmol/L	1.73 ± 1.23	1.63 ± 1.02	1.86 ± 1.45	< 0.001
HDL‐C, mmol/L	1.26 ± 0.34	1.29 ± 0.34	1.23 ± 0.33	< 0.001
LDL‐C, mmol/L	2.58 ± 0.81	2.57 ± 0.78	2.59 ± 0.86	0.558
Apo‐A1, g/L	1.15 ± 0.27	1.16 ± 0.28	1.13 ± 0.26	0.003
Apo‐B, g/L	0.82 ± 0.24	0.82 ± 0.23	0.83 ± 0.26	0.064

Abbreviations: Apo‐A1, apolipoprotein A1; Apo‐B, apolipoprotein B; BMI, body mass index; CAD, coronary artery disease; HDL‐C, high‐density lipoprotein cholesterol; LDL‐C, low‐density lipoprotein cholesterol; TC, total cholesterol; TG, triglycerides.

### 3.2 Distribution Frequencies of the *CYP2C19* Genotypes and Alleles in CAD Patients and Controls

There were 1567 (46.0%), 1287 (37.8%), 204 (6.0%), 270 (7.9%), 66 (1.9%), and 10 (0.3%) individuals with *CYP2C19* *1/*1, *1/*2, *1/*3, *2/*2, *2/*3, and *3/*3 genotype, respectively. There were 1567 (46.0%), 1491 (43.8%), and 346 (10.2%) individuals with *CYP2C19* EM, IM, and PM phenotype, respectively. The results of the Hardy–Weinberg equilibrium test showed that the *CYP2C19* genotypes in the controls (*χ* ^2^ = 4.320, *p* = 0.364) and CAD patients (*χ* ^2^ = 2.708, *p* = 0.608) confirmed to the Hardy–Weinberg equilibrium, respectively. The frequency of the *CYP2C19* *2/*2 genotype was higher (9.7% vs. 6.6%, *p* = 0.001), while the frequency of the *CYP2C19* *1/*1 genotype was lower (42.3% vs. 48.8%, *p* < 0.001) in the CAD patients than those in controls. The CAD patients had higher frequencies of the *2 allele (30.2% vs. 26.0%, *p* < 0.001), *3 allele (4.9% vs. 3.8%, *p* = 0.021) and lower frequency of *1 allele (64.8% vs. 70.2%, *p* < 0.001) than controls (Table 2).

**TABLE 2:** Distribution frequencies of *CYP2C19* genotypes and alleles in CAD patients and controls.

CYP2C19 phenotypes	*CYP2C19* genotypes/alleles	Total (*n* = 3404)	Controls (*n* = 1966)	CAD patients (*n* = 1438)	*χ* ^2^	*p*
	Genotypes					
Extensive metabolizer	*1/*1	1567 (46.0%)	959 (48.8%)	608 (42.3%)	14.117	< 0.001
Intermediate metabolizer	*1/*2	1287 (37.8%)	734 (37.3%)	553 (38.5%)	0.444	0.520
	*1/*3	204 (6.0%)	108 (5.5%)	96 (6.7%)	2.062	0.165
Poor metabolizer	*2/*2	270 (7.9%)	130 (6.6%)	140 (9.7%)	11.094	0.001
	*2/*3	66 (1.9%)	30 (1.5%)	36 (2.5%)	4.174	0.044
	*3/*3	10 (0.3%)	5 (0.3%)	5 (0.3%)	0.247	0.751
	Alleles					
	*1	4625 (67.9%)	2760 (70.2%)	1865 (64.8%)	21.795	< 0.001
	*2	1893 (27.8%)	1024 (26.0%)	869 (30.2%)	14.408	< 0.001
	*3	290 (4.3%)	148 (3.8%)	142 (4.9%)	5.608	0.021
	HWE (*χ* ^2^, *p*)	*χ* ^2^ = 5.459, *p* = 0.243	*χ* ^2^ = 4.320, *p* = 0.364	*χ* ^2^ = 2.708, *p* = 0.608		

Abbreviation: HWE, Hardy Weinberg Equilibrium.

### 3.3 Clinical Characteristics of Subjects Stratified by *CYP2C19* Phenotypes

**FIGURE 1:**
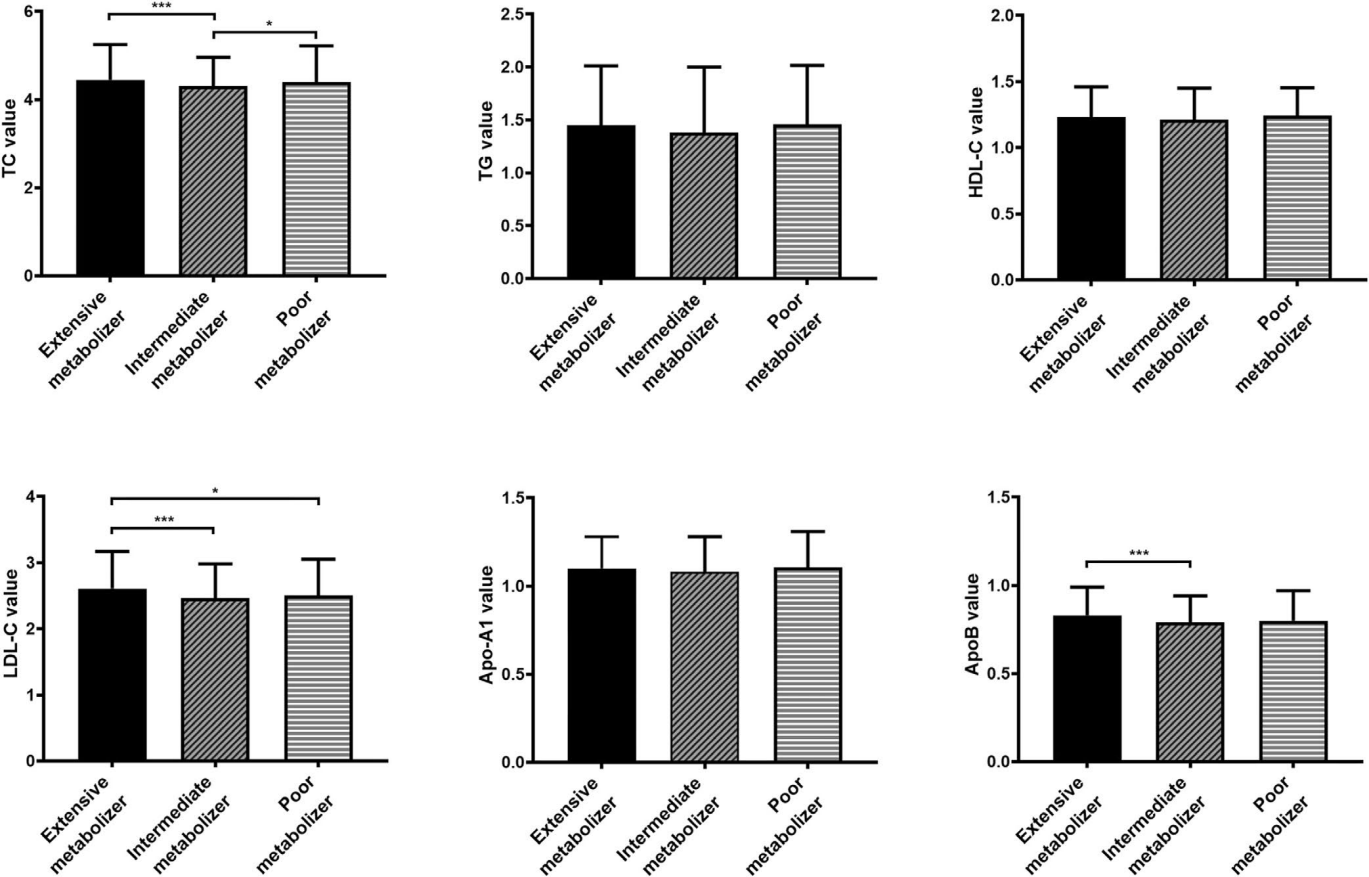
Comparison of TC, TG, HDL‐C, LDL‐C, Apo‐A1, and Apo‐B in subjects stratified by CYP2C19 phenotypes. Apo‐A1, apolipoprotein A1; Apo‐B, apolipoprotein B; HDL‐C, high‐density lipoprotein cholesterol; LDL‐C, low‐density lipoprotein cholesterol; TC, total cholesterol; TG, triglycerides. *p < 0.05, ***p < 0.001.

Clinical characteristics and serum lipid‐lipoprotein levels were compared among all subjects carrying different *CYP2C19* phenotypes. The proportion of female patients in the *CYP2C19* PM group was higher than that in the *CYP2C19* EM and IM groups (44.2% vs. 38.6% and 35.6%, *p* = 0.009). The proportion of BMI ≥ 24.0 kg/m^2^ in the *CYP2C19* PM group was lower than that in the *CYP2C19* EM and IM groups (41.9% vs. 50.9% and 49.8%, *p* = 0.044) (Table 3). The patients with the *CYP2C19* IM phenotype had a lower TC level (4.36 ± 1.04 mmol/L vs. 4.56 ± 1.17 mmol/L, *p* < 0.001) and LDL‐C (2.49 ± 0.72 vs. 2.67 ± 0.88 mmol/L, *p* < 0.001) than those with the *CYP2C19* EM phenotype. The patients with the *CYP2C19* IM phenotype had a lower TC level (4.36 ± 1.04 mmol/L vs. 4.55 ± 1.41 mmol/L, *p* < 0.001) than those with the *CYP2C19* PM phenotype (Figure 1).

**TABLE 3:** Clinical characteristics of subjects stratified by *CYP2C19* phenotypes.

Variables	Extensive metabolizer (*n* = 1567)	Intermediate metabolizer (*n* = 1491)	Poor metabolizer (*n* = 346)	*p*
Gender				
Male, *n* (%)	962 (61.4%)	960 (64.4%)	193 (55.8%)	0.009 (*χ* ^2^ = 9.519)
Female, *n* (%)	605 (38.6%)	531 (35.6%)	153 (44.2%)	
Age, years				
< 65, *n* (%)	539 (34.4%)	509 (34.1%)	128 (37.0%)	0.595 (*χ* ^2^ = 1.042)
≥ 65, *n* (%)	1028 (65.6%)	982 (65.9%)	218 (63.0%)	
BMI (kg/m^2^)				
< 18.5	45 (2.9%)	51 (3.4%)	13 (3.8%)	0.044 (*χ* ^2^ = 9.775)
18.5–23.9	725 (46.3%)	698 (46.8%)	188 (54.3%)	
≥ 24.0	797 (50.9%)	742 (49.8%)	145 (41.9%)	
History of smoking				
No	1244 (79.4%)	1166 (78.2%)	284 (82.1%)	0.264 (*χ* ^2^ = 2.665)
Yes	323 (20.6%)	325 (21.8%)	62 (17.9%)	
History of alcohol consumption				
No	1509 (96.3%)	1429 (95.8%)	335 (96.8%)	0.662 (*χ* ^2^ = 0.897)
Yes	58 (3.7%)	62 (4.2%)	11 (3.2%)	
Diabetes mellitus				
No	1103 (70.4%)	1064 (71.4%)	236 (68.2%)	0.498 (*χ* ^2^ = 1.404)
Yes	464 (29.6%)	427 (28.6%)	110 (31.8%)	

Abbreviations: BMI, body mass index; CAD, coronary artery disease.

### 3.4 Clinical Characteristics of Subjects Stratified by *CYP2C19* Phenotypes in CAD Patients With Different Ages

In both CAD patients with age < 65 years and age ≥ 65 years there was no significant difference in distributions of gender and BMI, and proportions of history of smoking, history of alcohol consumption, and diabetes mellitus, and serum lipid‐lipoprotein levels among different CYP2C19 phenotypes, respectively (all *p* > 0.05) (Table 4).

**TABLE 4:** Clinical characteristics of subjects stratified by *CYP2C19* phenotypes in CAD patients with different ages.

Variables	Age < 65 years (*n* = 508)				Age ≥ 65 years (*n* = 930)			
	Extensive metabolizer (*n* = 221)	Intermediate metabolizer (*n* = 219)	Poor metabolizer (*n* = 68)	*p*	Extensive metabolizer (*n* = 387)	Intermediate metabolizer (*n* = 430)	Poor metabolizer (*n* = 113)	*p*
Gender								
Male, *n* (%)	161 (72.9%)	158 (72.1%)	42 (61.8%)	0.190 (*χ* ^2^ = 3.327)	218 (56.3%)	248 (57.7%)	56 (49.6%)	0.301 (*χ* ^2^ = 2.405)
Female, *n* (%)	60 (27.1%)	61 (27.9%)	26 (38.2%)		169 (43.7%)	182 (42.3%)	57 (50.4%)	
BMI (kg/m^2^)								
< 18.5	2 (0.9%)	2 (0.9%)	2 (2.9%)	0.554 (*χ* ^2^ = 3.014)	15 (3.9%)	17 (4.0%)	4 (3.5%)	0.193 (*χ* ^2^ = 6.056)
18.5–23.9	79 (35.7%)	84 (38.4%)	28 (41.2%)		169 (43.7%)	203 (47.2%)	64 (56.6%)	
≥ 24.0	140 (63.3%)	133 (60.7%)	38 (55.9%)		203 (52.5%)	210 (48.8%)	45 (39.8%)	
History of smoking								
No	149 (67.4%)	168 (76.7%)	48 (70.6%)	0.090 (*χ* ^2^ = 4.757)	314 (81.1%)	338 (78.6%)	98 (86.7%)	0.149 (*χ* ^2^ = 3.884)
Yes	72 (32.6%)	51 (23.3%)	20 (29.4%)		73 (18.9%)	92 (21.4%)	15 (13.3%)	
History of alcohol consumption								
No	208 (94.1%)	204 (93.2%)	63 (92.6%)	0.881 (*χ* ^2^ = 0.264)	371 (95.9%)	409 (95.1%)	109 (96.5%)	0.751 (*χ* ^2^ = 0.502)
Yes	13 (5.9%)	15 (6.8%)	5 (7.4%)		16 (4.1%)	21 (4.9%)	4 (3.5%)	
Diabetes mellitus								
No	155 (70.1%)	164 (74.9%)	43 (63.2%)	0.157 (*χ* ^2^ = 3.680)	274 (70.8%)	309 (71.9%)	71 (62.8%)	0.170 (*χ* ^2^ = 3.568)
Yes	66 (29.9%)	55 (25.1%)	25 (36.8%)		113 (29.2%)	121 (28.1%)	42 (37.2%)	
Serum lipid‐lipoprotein levels								
TC, mmol/L	4.73 ± 1.29	4.74 ± 1.33	5.07 ± 2.30	0.220	4.51 ± 1.15	4.48 ± 1.12	4.51 ± 1.12	0.891
TG, mmol/L	2.03 ± 1.40	2.18 ± 2.03	2.10 ± 2.14	0.713	1.76 ± 1.21	1.70 ± 1.26	1.65 ± 0.94	0.665
HDL‐C, mmol/L	1.18 ± 0.32	1.16 ± 0.28	1.22 ± 0.34	0.436	1.26 ± 0.35	1.25 ± 0.34	1.25 ± 0.31	0.899
LDL‐C, mmol/L	2.70 ± 0.93	2.67 ± 0.91	2.85 ± 0.94	0.389	2.55 ± 0.85	2.50 ± 0.80	2.51 ± 0.82	0.726
Apo‐A1, g/L	1.14 ± 0.25	1.10 ± 0.24	1.15 ± 0.27	0.216	1.14 ± 0.27	1.13 ± 0.28	1.13 ± 0.25	0.891
Apo‐B, g/L	0.88 ± 0.28	0.87 ± 0.27	0.88 ± 0.26	0.982	0.81 ± 0.24	0.81 ± 0.24	0.82 ± 0.24	0.843

Abbreviations: Apo‐A1, apolipoprotein A1; Apo‐B, apolipoprotein B; BMI, body mass index; CAD, coronary artery disease; HDL‐C, high‐density lipoprotein cholesterol; LDL‐C, low‐density lipoprotein cholesterol; TC, total cholesterol; TG, triglycerides.

### 3.5 Logistic Regression Analysis of Risk Factors of CAD

The results of univariate analysis showed that *CYP2C19* IM + PM phenotypes (IM + PM phenotypes vs. EM phenotype, odds ratio (OR): 1.300, 95% confidence interval (CI): 1.134–1.491, *p* < 0.001), BMI ≥ 24.0 kg/m^2^ (BMI ≥ 24.0 kg/m^2^ vs. BMI 18.5–23.9 kg/m^2^, OR: 1.319, 95% CI: 1.148–1.515, *p* < 0.001), history of smoking (yes vs. no, OR: 1.182 95% CI: 1.001–1.396, *p* = 0.049), and history of alcoholism (yes vs. no, OR: 1.817 95% CI: 1.227–2.584, *p* = 0.001) were significantly associated with CAD. Multivariate logistic regression analysis showed that *CYP2C19* IM + PM phenotypes (IM + PM phenotypes vs. EM phenotype, OR: 1.314, 95% CI: 1.145–1.508, *p* < 0.001), BMI ≥ 24.0 kg/m^2^ (BMI ≥ 24.0 kg/m^2^ vs. BMI 18.5–23.9 kg/m^2^, OR: 1.364, 95% CI: 1.184–1.571, *p* < 0.001), and history of alcoholism (yes vs. no, OR: 1.761 95% CI: 1.211–2.559, *p* = 0.003) were independent risk factors for CAD in hypertensive patients (Table 5).

**TABLE 5:** Logistic regression analysis of risk factors of CAD.

Variables	Univariate OR (95% CI)	*p*	Multivariate OR (95% CI)	*p*
Gender (female/male)	1.055 (0.917–1.214)	0.454	1.162 (0.995–1.357)	0.058
Age (≥ 65/< 65, years)	0.942 (0.817–1.087)	0.414	1.004 (0.867–1.162)	0.956
BMI (kg/m^2^)				
18.5–23.9	1.000 (reference)	—	1.000 (reference)	—
< 18.5	0.984 (0.660–1.465)	0.936	0.958 (0.640–1.433)	0.835
≥ 24.0	1.319 (1.148–1.515)	< 0.001	1.364 (1.184–1.571)	< 0.001
History of smoking (yes/no)	1.182 (1.001–1.396)	0.049	1.190 (0.982–1.442)	0.076
History of alcoholism (yes/no)	1.817 (1.277–2.584)	0.001	1.761 (1.211–2.559)	0.003
Diabetes mellitus (yes/no)	0.995 (0.857–1.155)	0.947	0.981 (0.843–1.141)	0.800
*CYP2C19* phenotypes				
Extensive metabolizer	1.000 (reference)	—	1.000 (reference)	—
Intermediate metabolizer	1.216 (1.052–1.404)	0.008	1.222 (1.057–1.413)	0.007
Poor metabolizer	1.730 (1.369–2.187)	< 0.001	1.790 (1.413–2.266)	< 0.001
Intermediate metabolizer + poor metabolizer	1.300 (1.134–1.491)	< 0.001	1.314 (1.145–1.508)	< 0.001

Abbreviation: BMI, body mass index.

## 4 Discussion

CAD is one of the common diseases in cardiovascular medicine. In recent years, the incidence of CAD is on the rise with the increasing aging of our population [28]. The pathogenesis of CAD is complex, and most scholars believe that under the action of various major risk factors, endothelium‐lining injury and inflammatory fibrosis occur, leading to the formation of atherosclerotic lesions [29]. In pathological conditions, long‐term elevated blood pressure leads to higher blood flow impact pressure on arterial endothelial cells, resulting in intimal injury and LDL‐C entering the artery wall, which stimulates smooth muscle cell proliferation, activates inflammation, and leads to lipid deposition and mural thrombosis [30]. In addition, the increase in blood pressure can make the cerebral cortex in a long‐term excitatory state, and the catecholamines released by the sympathetic nerve can directly damage the arterial blood wall [31]. At the same time, the body's enhanced sensitivity to the sympathetic nerve will cause vascular contraction, cause myocardial ischemia and hypoxia, and lead to CAD [32].

More than half of CVDs in China are related to hypertension. For patients with hypertension, the blood pressure level is high for a long time, and the pressure increases accordingly, which then causes damage to vascular endothelial function and causes left ventricular hypertrophy, atherosclerosis, arrhythmia, and other symptoms under the action of vasoactive substances. It may also lead to the occurrence of CAD. Studies have shown that hypertension is prone to co‐exist with metabolic diseases such as obesity, abnormal blood sugar, and dyslipidemia, and these metabolic diseases often become an important cause of the occurrence and death of CVDs [33]. In patients with hypertension, the risk factors for CAD remain unclear. Therefore, it is of great significance to identify the risk of CAD in hypertensive patients.

As an indicator of the degree of obesity, studies have found that when BMI is maintained in the normal range, the occurrence of CAD events can be reduced [34]. Chen et al. found that BMI ≥ 24.0 kg/m^2^ was an independent risk factor for type 2 diabetes mellitus (T2DM) complicated with CAD [35]. In addition, BMI is associated with long‐term prognosis and risk of death from CAD [36, 37]. BMI is different in population due to gender, age, and race, and has certain limitations in risk prediction. Therefore, comprehensive attention should be paid to the role of some risk factors in CAD susceptibility in people with hypertension.

In terms of the relationship between alcohol consumption and CAD, different studies have different results. Hypertensive patients with a history of alcohol consumption have a higher risk of CAD [38]. Chen et al. found that a history of alcohol consumption was an independent risk factor for T2DM complicated with CAD [35]. The difference between a history of alcohol consumption and the risk of cerebral infarction and CAD was not statistically significant [21]. In addition, occasional alcohol consumption is associated with less severe cardiovascular damage [39]. In this study, a history of alcoholism was an independent risk factor for CAD in hypertensive patients.

Hypertensive people with a history of smoking have a higher risk of major cardiovascular events. Among smoking‐related deaths, CVD accounts for approximately one third of cases worldwide [40]. There is a dose–response relationship between the number of cigarettes smoked per day and CVD, and even moderate amounts of daily smoking increase the risk of myocardial infarction [41]. The mechanism of the potential association between tobacco and CVD may be due to the fact that tobacco smoke contains thousands of chemicals (including nicotine, carbon monoxide and so on), which promote the development of CVD by increasing heart rate, myocardial contractility, inflammation, endothelial damage, and thrombosis, and lowering HDL‐C levels [42]. In this study, univariate logistic regression analysis showed that smoking was a risk factor for CAD in the hypertensive population, but multivariate logistic regression analysis did not obtain this result. However, in any case, health education should be strengthened for hypertensive patients and smoking cessation intervention should be carried out to reduce the risk of CAD.

The role of CYP2C19 in cardiovascular function may involve some biological processes. For example, CYP2C19‐catalyzed arachidonic acid (AA) endoderm hyperpolarization factor (EDHF) metabolites play an antihypertensive, anti‐inflammatory, and anticoagulant role by activating potassium ion and calcium ion channels, inhibiting platelet aggregation, inhibiting white blood cell adhesion to the blood vessel wall, and reducing the expression of vascular cell adhesion molecules [43]. CYP2C19 biotransforms 5‐hydroxytryptamine (5‐HT) to produce hydroxylamine, which is converted to nitric oxide in the presence of catalase, and this process can relax the pre‐contracted aortic ring in vitro [44]. To our knowledge, there have been few studies on the relationship between *CYP2C19* polymorphisms and CAD susceptibility. CYP2C19*3 polymorphism is a risk factor for CAD in the Chinese Uyghur population [45]. CYP2C19 PM was an independent risk factor of CAD in Japanese women [25]. CYP2C19 EM phenotype was dominant in CAD patients among a Chinese population [21]. Akasaka et al. found that female sex, smoking, and hypertension were associated with coronary microvascular disorder (CMVD), and CYP2C19 PM was a predictive factor for CMVD in the female population [46].

In this study, *CYP2C19* loss‐of‐function, BMI ≥ 24.0 kg/m^2^, and history of alcoholism were independent risk factors for CAD in hypertensive patients. It means that hypertensive patients who are obese, have a history of alcoholism, and carry the CYP2C19 IM or PM phenotype need to be aware of the risk of developing CAD. This study is the first to report on *CYP2C19* gene polymorphisms and CAD susceptibility in hypertensive patients. There are some shortcomings in this study. First, as a retrospective study, this study only collected some possible risk factor indicators from the medical record system of the subjects and did not include other possible disease‐influencing factors, such as dietary and living habits and concomitant medications. Second, the subjects of this study come from a single medical institution, and the selection of the population may be biased, so the universality of the results of this study in the population needs to be further verified. Third, this study did not classify hypertensive patients and analyze the relationship between CYP2C19 gene polymorphisms and CAD risk in patients with different hypertension grades.

## 5 Conclusions

In summary, *CYP2C19* loss‐of‐function, BMI ≥ 24.0 kg/m^2^, and history of alcoholism were independent risk factors for CAD in hypertensive patients. It means that hypertensive patients who are obese, have a history of alcoholism, and carried the CYP2C19 IM or PM phenotype need to be aware of the risk of developing CAD.

## Author Contributions

Guoliang Wei and Hui Rao designed the study. Guoliang Wei, Bin Li, Hao Wang, Wenhao Chen, Kehui Chen, Weihong Wang, Shen Wang, Hui Zeng, Yuanliang Liu, Yue Zeng, and Hui Rao collected clinical data. Guoliang Wei and Hui Rao analyzed the data. Guoliang Wei prepared the manuscript. All authors were responsible for critical revisions, and all authors read and approved the final version of this work.

## Conflicts of Interest

The authors declare no conflicts of interest.

## Data Availability

The data that support the findings of this study are available on request from the corresponding author.
